# Seamless vital sign monitoring with fiber optic sensing technology: validating the sitting SMART MAT for community health in static and mobile conditions

**DOI:** 10.3389/fphys.2026.1875461

**Published:** 2026-07-23

**Authors:** Yan Yi Sim, Darryl J. Q. Chan, Yong Xiang Phang, Marc S. L. Liem, Meredith T. Yeung

**Affiliations:** Health and Social Sciences Cluster, Singapore Institute of Technology, Singapore, Singapore

**Keywords:** ballistocardiography (BCG), community health, fiber-optic sensors, non-invasive monitoring, validation study, vital sign monitoring, wheelchair users

## Abstract

**Background:**

Continuous vital sign monitoring is critical but often labor-intensive. Fiberoptic technologies, like the SMART MAT, offer non-invasive assessment. Adapting this for a seated posture addresses clinical needs by promoting safe patient mobilization and reducing complications associated with prolonged bed rest.

**Objectives:**

To validate the accuracy of the sitting SMART MAT for respiratory rate (RR), heart rate (HR), oxygen saturation (SpO_2_), and systolic and diastolic blood pressure (SBP/DBP) against established clinical reference standards.

**Methods:**

This cross-sectional study compared sitting SMART MAT readings with clinical reference measurements under stationary and mobile wheelchair conditions. Accuracy was assessed using Mean Absolute Error (MAE), Mean Absolute Percentage Error (MAPE), Bland-Altman Limits of Agreement (LoA), repeated-measures ANOVA, and effect size.

**Results:**

Data from 130 participants were analyzed. HR readings demonstrated high accuracy (MAE = 3.67–4.81 bpm) with tight LoA. RR error was minimal (MAE = 2.85–2.86 breaths/min) statically but increased during motion (MAE = 5.03–5.21 breaths/min). SpO2 remained highly stable (MAE = 1.08–1.21%), well within standard clinical tolerance. SBP showed solid central accuracy (MAE = 6.48–7.51 mmHg) but wider LoA occasionally exceeded the ±5 mmHg threshold, functioning best as a continuous trend monitor. DBP variance was negligible (MAE = 4.65–5.46 mmHg).

**Conclusion:**

The current SMART MAT is a viable, integrated multimodal platform. HR, RR, and DBP are comparable to clinical reference standards, while SBP requires careful interpretation. By accommodating a functional sitting posture, the contactless system reduces immobility-related complications and demonstrates high feasibility for continuous, real-world monitoring.

## 1 Introduction

Continuous monitoring of vital signs is fundamental for early detection of clinical deterioration and the timely execution of medical interventions. Traditionally consisting of body temperature, heart rate (HR) or pulse rate (PR), blood pressure (BP), oxygen saturation (SpO_2_), and respiratory rate (RR) (Brekke et al., 2019), they serve as indicators for fluctuations in physiological function or responses to medical treatment (Elliott, 2021), allowing healthcare professionals to recognize patient decline. Ensuring accurate measurements is critical for preventing adverse events in acute settings (Mok et al., 2015; Chalari et al., 2012). Although traditional physiological monitoring methods are highly accurate, they are often labor-intensive, obtrusive, and inefficient for long-term use in community and outpatient settings. Equipment handling remains a persistent, laborious issue in fast-paced healthcare environments (Dall'ora et al., 2020), and inadequate monitoring is inevitable (Smith et al., 2017), potentially allowing deterioration to go unnoticed. Recognition of fluctuations in vital signs is an established factor in identifying and managing patient deterioration (Liu et al., 2024). Consequently, the healthcare industry has a strong demand for more efficient methods of continuous patient surveillance. To address these limitations, emerging fiber-optic sensor technologies have been developed. Adopting the principles of fiberoptic sensors, changes in body vibration evoked by breathing and cardiac rhythms cause variations in reflected wavelengths (Nedoma et al., 2017). These sensors are lightweight, biocompatible, and immune to electromagnetic interference (Gan et al., 2024). This has led to the development of the SMART MAT, an integrated multi-modal platform that can be embedded into textiles and seating surfaces for contactless monitoring (Fajkus et al., 2017). Using techniques such as ballistocardiography and pressure-variation analysis, the non-contact fiber-optic sensors embedded in the cushion estimate HR and RR without requiring the patient to wear restrictive devices. To provide a comprehensive surveillance profile, this hybrid system also integrates conventional tethered peripheral sensors to capture BP and SpO_2_. This configuration serves as a vital translational step toward a fully passive system, aggregating data centrally on an external dashboard to reduce the need for independent device deployments by healthcare workers. Previously, a bed-mattress version of the SMART MAT was designed for hospitalized or bed-bound individuals and demonstrated reasonable accuracy relative to established clinical reference standards (Ng et al., 2025). However, the mat’s size does not accommodate patients who are chair-bound when seated in geriatric chairs or wheelchairs. While the vast majority of existing contactless monitoring systems are designed exclusively for mattresses, prolonged bed rest in elderly and rehabilitative populations is strongly associated with physical deconditioning, pressure injuries, and pulmonary complications (Dittmer and Teasell, 1993). Thus, a sitting version of the SMART MAT addresses a critical clinical and social gap by providing continuous surveillance while encouraging patients to safely mobilize to an upright posture. Furthermore, sitting postures produce physiological variations compared to the supine positions (Jones and Dean, 2004). Thus, the SMART MAT sitting version was conceptualized to address this gap. Despite the potential of integrated monitoring, validation under varying environmental and physiological conditions remains essential. Considering the fiberoptic catchment and placement modifications, as well as the technology’s novelty, the practicality of the SMART MAT for sitting in both static and mobile settings must be assessed before implementation in healthcare environments. Therefore, this study aims to evaluate the accuracy and validity of the sitting SMART MAT against current clinical reference standard measurements. It is hypothesized that these integrated SMART MAT measurements are comparable to established clinical methods in healthy individuals.

## 2 Methodology

### 2.1 Study design and reporting

This cross-sectional convenience sampling study was conducted and reported according to the Strengthening the Reporting of Observational Studies in Epidemiology (STROBE) guidelines (Von Elm et al., 2007). The University Institute Review Board granted approval (Approval number RECAS-0493), and data collection took place from January to October 2025 on the university premises. All participants provided written informed consent before taking part in the study; only anonymized data were analyzed, with access restricted to the researchers. Four undergraduate physiotherapy students proficient in vital signs assessment collected data, with all data points measured on the same day for each participant; no follow-ups were needed.

### 2.2 Participants and sample size calculation

Convenience sampling recruited participants from the local community. Inclusion criteria for the study were healthy adults aged 21 and above. The exclusion criteria comprised the following: (1) individuals unable to partake in vital signs measurement, including a history of mastectomy with radiation therapy, regardless of axillary lymph node dissection, as the increased pressure during BP monitoring may increase the risk of lymphoedema (Bryant et al., 2016); (2) arteriovenous fistula/dialysis shunt due to the risk of thrombosis formation and infection by BP measurement over the fistula site (Schwab et al., 2001); (3) known allergy to the adhesive of ECG leads (Kellogg et al., 2022); (4) known arrhythmias, such as atrial fibrillation and atrial flutter, which can lead to rhythm irregularities that could cause inaccuracies in recorded values (Pagonas et al., 2013).

Sample size calculations were based on preliminary data from the first 50 pilot measurements comparing the sitting SMART MAT with clinical reference standard measurements of HR, RR, BP, and SpO_2_. A selected sample size of 100 demonstrated >95% power for HR [minimal clinically important differences (MCID) = 5 beats/min], >99% power for SBP, 99.99% power for DBP (MCID for SBP and DBP = 5 mmHg), >99% power for RR (MCID = 2 breaths/min), and 99.9% power for SpO_2_ (MCID ±2% margin). Hence, a minimum sample size of n = 100 was deemed sufficient, assuming non-normality persisted, with an additional 30% to account for potential exclusion. This sample size is in line with regulatory standards for the study.

### 2.3 Procedure

The recruited participants were provided with standardized pre-study instructions as follows: (1) wear loose and comfortable clothing (e.g., t-shirt with short sleeves) to ease the placement of blood pressure monitoring and electrode placements on the chest and legs; (2) remove any nail polish; (3) avoid lotion or other skincare products on the body; (4) avoid consuming caffeinated beverages (e.g., tea, coffee and energy drinks) and alcohol 24 hours before the session; (5) avoid engaging in vigorous activity one hour before the session; (6) avoid consuming food and beverages thirty minutes before the session; (7) empty bladder and bowel before data collection, if necessary; (8) remove footwear, socks, and/or accessories during body composition measurement, and; (9) during the study, sit on a chair and minimize movement and/or avoid speaking for twenty minutes while vital signs are monitored at various time intervals.

Two researchers facilitated each session. One researcher obtained written informed consent, while the second collected anthropometric data: including age, gender, body mass index (BMI), and body composition, using a stadiometer and body composition analyzer. Participants were seated on a standardized wheelchair comprising a 5-centimeter (cm) height seat cushion, with the SMART MAT [Measured 310 millimeters (mm) x 280 mm x 5mm] placed beneath the cushion (**Supplementary Figure 1**). **Supplementary Figure 2** illustrates the form factor of the sitting SMART MAT. The first investigator attached SMART MAT-paired devices (an electronic BP cuff and a pulse oximeter) to the participant’s right arm. Concurrently, the second investigator applied clinical reference standard devices [manual sphygmomanometer, electrocardiogram (ECG), pulse oximeter] on the left side (**Supplementary Figure 3**). The SMART MAT would be calibrated for 5 minutes prior to data collection.

Data were collected over 20 minutes: the first 10 minutes were conducted with the wheelchair stationary, followed by 10 minutes of active movement (the wheelchair was pushed back and forth along a flat walking path). HR and BP were recorded at minutes 3, 8, 13, and 18; RR and SpO2 were recorded at minutes 5, 10, 15, and 20 (**Supplementary Figure 4**). Researchers logged readings simultaneously but separately for both systems. Following the 20-minute protocol, participants were observed for 5 minutes for any adverse reactions. The total session duration was approximately 30 minutes.

### 2.4 Instrumentation

#### 2.4.1 Anthropometric measurements

Anthropometric data, including weight, muscle mass, total body water, and fat-free mass, were acquired using the Tanita MC-980Uplus Multi-Frequency Segmental Body Composition Analyzer (Tanita, n.d). Stature was determined using the Seca 213 Portable Stadiometer (Seca, n.d), with measurements recorded to the nearest 1 millimeter (mm).

#### 2.4.2 Clinical reference standard devices

HR and electrical cardiac activity were obtained using the Schiller CardioVit AT-1 G2 Electrocardiogram (Schiller, n.d). Manual BP measurements were obtained using the Bokang LCD Mercury-Free Sphygmomanometer (Bokang, n.d), with a capacity for measurements up to 300 mmHg. Peripheral oxygen saturation (SpO2) was monitored using the Nellcor™ Portable Patient Monitoring System (Medtronic, n.d.). This device was selected for its proven reliability in challenging monitoring conditions, including signal interference and patient movement.

#### 2.4.3 SMART MAT technology

The current pre-production version of the sitting SMART MAT is similar to the previous version (Ng et al., 2025), but with a scaled-down form factor. It is an integrated monitoring system that utilizes patented multimode fiber-optic technology (Patent 10201404690R, Chen et al., 2014) to continuously assess HR and RR via contactless mechanisms. The sensor, constructed from water-resistant neoprene, encapsulates fiberoptics within a mechanically structured layer. This configuration induces pressure-sensitive microbending, facilitated by interwoven polyester layers that act as fiber-mode converters, modulating light intensity in direct response to cardiac- and respiratory-induced thoracic displacements.

The hardware architecture comprises a light source, a fiber coupler, a mirror, and a photodetector. HR detection is underpinned by a multi-channel ballistocardiography (BCG) algorithm (Patent 11201703737Q, Zhu et al., 2017), which isolates recoil forces generated by cardiac ejection of blood. To mitigate motion artifacts and address the non-stationary nature of BCG signals, the system employs frequency-domain transformations, dynamic channel selection, and cepstrum smoothing to derive robust estimates of heartbeat intervals. Concurrently, RR is determined via pressure variation analysis of cyclic chest displacement. Advanced signal processing techniques, including peak detection, Fast Fourier Transform (FFT), and wavelet analysis, extract these physiological parameters for real-time visualization and automated threshold-based alarms. This passive-sensing matrix, with a non-wearable, compact design, enables seamless integration into chairs or beds, maximizing both user comfort and clinical utility. To validate the comprehensive physiological profile of the sitting SMART MAT, peripheral data were collected using the Jumper Electronic Blood Pressure Monitor (Jumper, n.d.-a) and the Jumper Fingertip Pulse Oximeter (Jumper, n.d.-b). The blood pressure monitor uses oscillometric technology for automated, non-invasive assessment, while the pulse oximeter employs photoelectric oximetry to monitor SpO_2_ levels. Both devices adhere to the accuracy and safety standards established by the Health Sciences Authority of Singapore, the European Union, and the United States Food and Drug Administration. Processed physiological parameters and chair occupancy status are transmitted wirelessly, allowing users and clinicians to view real-time vital displacements via a dedicated application on linked smart devices. For portability and continuous operation, the system is powered by an external battery pack that requires recharging approximately once per month, using a capacity of 10,000 mAh under standard operating conditions.

### 2.5 Statistical analysis

JASP (version 0.95.4) and Microsoft Excel were used for all statistical and data analyses. The Kolmogorov-Smirnov test determined the data’s normality. Non-parametric statistical tests provided a standardized framework for the analysis. Differences between demographics and anthropometric variables between female and male participants were analyzed using the Mann-Whitney U test. Descriptive statistics are presented as mean ± [standard deviation (SD)] and median [Interquartile range (IQR)]. To quantify the magnitude of the differences between groups, effect size (r-value) was calculated by dividing the Z-score by the square root of the total sample size (r): $r=\frac{z}{\sqrt{N}}$. Effect sizes (r) were interpreted as small (0.1), medium (0.3), or large (0.5). Repeated-Measures Analysis of Variance (ANOVA) and effect sizes, measured as Eta-squared (η^2^), were used to compare SMART MAT with the clinical reference standards. While normality testing indicated deviations in certain parameters, the repeated-measures ANOVA was retained, as it is highly robust to violations of the normality assumption, given the large sample size (n ≥ 100) and the Central Limit Theorem. These ANOVA results serve as secondary sensitivity analyses to evaluate temporal stability. Effect size (η^2^) was interpreted according to established benchmarks: small (≥ 0.01), medium (≥ 0.06), and large (≥ 0.14) (Peres, 2026). A Bonferroni post hoc test (*p*Bonf) at each data point mitigates the family-wise error rate (Armstrong, 2014). To determine clinical agreement, the Mean Absolute Error (MAE) was calculated in standard clinical units across all parameters. Mean Absolute Percentage Error (MAPE) additionally evaluated the SMART MAT’s forecasting accuracy relative to the clinical reference standard. MAPE values indicated the following accuracy levels: highly accurate (<10%), good (10%-20%), reasonable (20%-50%), and inaccurate (>50%) (Montaño Moreno et al., 2013). Furthermore, Bland-Altman plots assessed the limits of agreement (LoA) between the sitting SMART MAT and clinical reference standards. In these plots, a positive mean difference signifies a tendency for the SMART MAT to overestimate measurements, whereas a negative mean difference signifies underestimation (Giavarina, 2015). All statistical tests utilized a 95% confidence level, with p < 0.05 defining statistical significance.

## 3 Results

**Figure 1:**
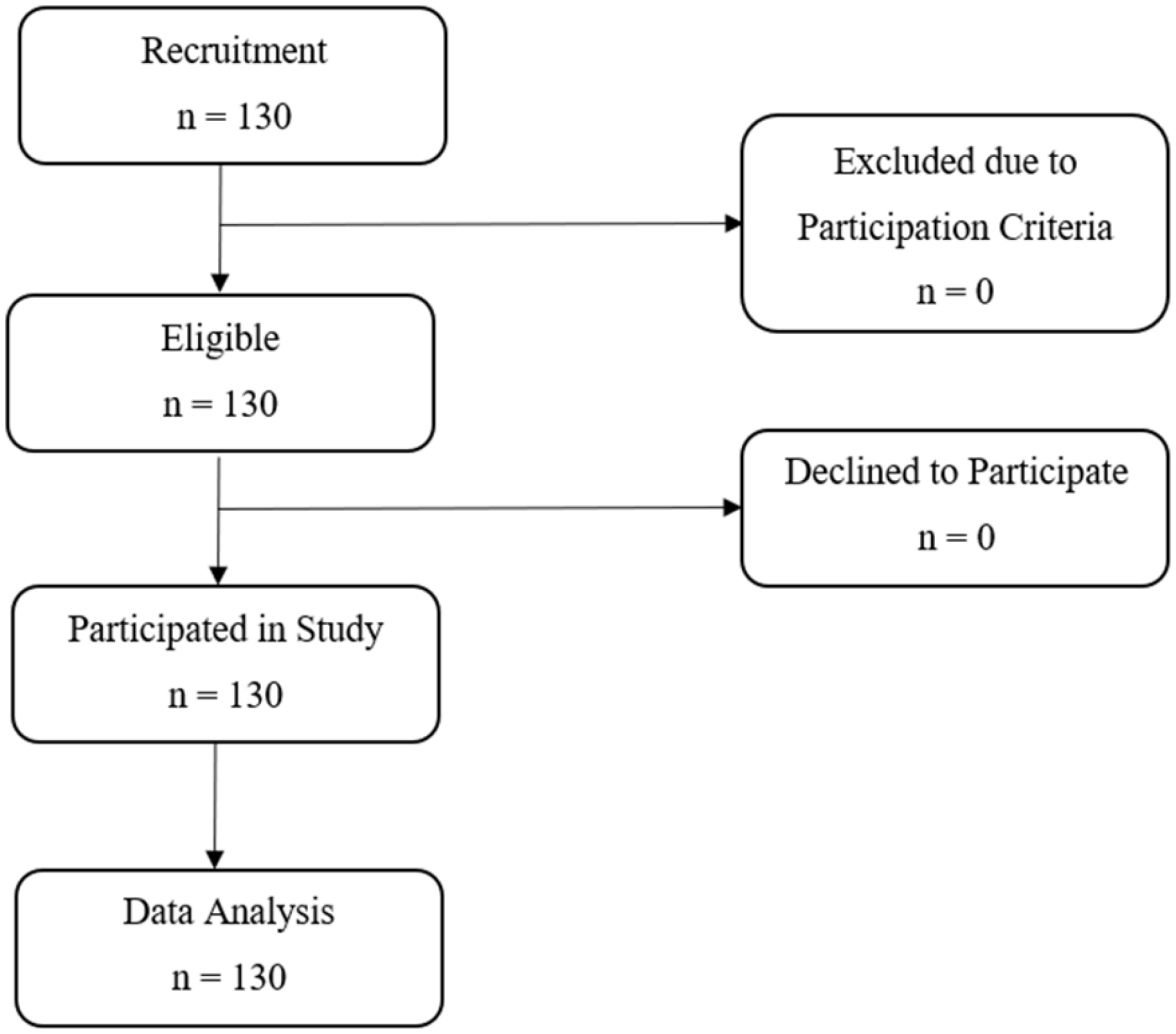
Participant recruitment flowchart.

**Figure 1** presents the flowchart for participant recruitment. 130 participants (female: 64; male: 66) were recruited, with no decline or withdrawals from the study. All the data collected were analyzed without exclusion. **Table 1** summarizes the participants’ demographics and anthropometrics. The study population primarily comprised young adults in their late twenties and thirties. Anthropometric analysis revealed significant gender-based dimorphism (p < 0.001): males exhibited higher mean height, weight, muscle mass, fat-free mass, and total body water, whereas females displayed significantly higher body fat percentages. Despite these variations, BMI did not differ significantly between genders (p=0.066), though a higher proportion of females fell within the healthy Asian BMI range.

**Table 1:** Demographics and anthropometric data of the participants.

Characteristic	Participant gender				Mann-Whitney U Test		Effect size (r)
	Female (n = 64)		Male (n = 66)		U	p-value	r-value
	Mean ± SD	Median (IQR)	Mean ± SD	Median (IQR)			
Age (years)	38.4 ± 15.7	34 (23.8-51.0)	32.7 ± 11.9	26 (25.0-27.0)	2360	0.249	0.117
Height (m)	1.60 ± 0.06	1.59 (1.55-1.64)	1.72 ± 0.08	1.72 (1.68-1.78)	470	<0.001*	-0.778
Weight (kg)	58.3 ± 10.9	55.9 (50.3-65.5)	70.6 ± 11.5	71.1 (62.7-76.5)	886	<0.001*	-0.581
Body Mass Index (kg/m^2^)	22.8 ± 3.48	21.7 (19.9-24.9)	23.8 ± 3.60	23.2 (21.4-25.8)	1716	0.066	-0.188
Body Fat Percentage (%)	31.2 ± 7.13	31.4 (26.0-35.9)	19.9 ± 7.02	20.1 (14.9-24.1)	3695	<0.001*	0.750
Muscle Mass (kg)	37.2 ± 3.90	37.5 (34.5-39.3)	53.2 ± 6.54	53.4 (49.6-57.2)	100	<0.001*	-0.953
Fat-Free Mass (kg)	39.1 ± 4.88	39.4 (36.2-41.6)	56.1 ± 6.88	56.3 (52.3-60.3)	113	<0.001*	-0.947
Total Body Water (%)	48.9 ± 4.49	48.4 (45.4-52.5)	54.0 ± 6.35	53.6 (50.7-58.1)	1033	<0.001*	-0.511

n, sample size; SD, standard deviation; IQR, interquartile range 25%-75%; m, meters; kg, kilograms. *U*, Mann-Whitney U test statistic; r, effect size; *: denotes *p* <.05, which represents statistical significance.

**Figure 2:**
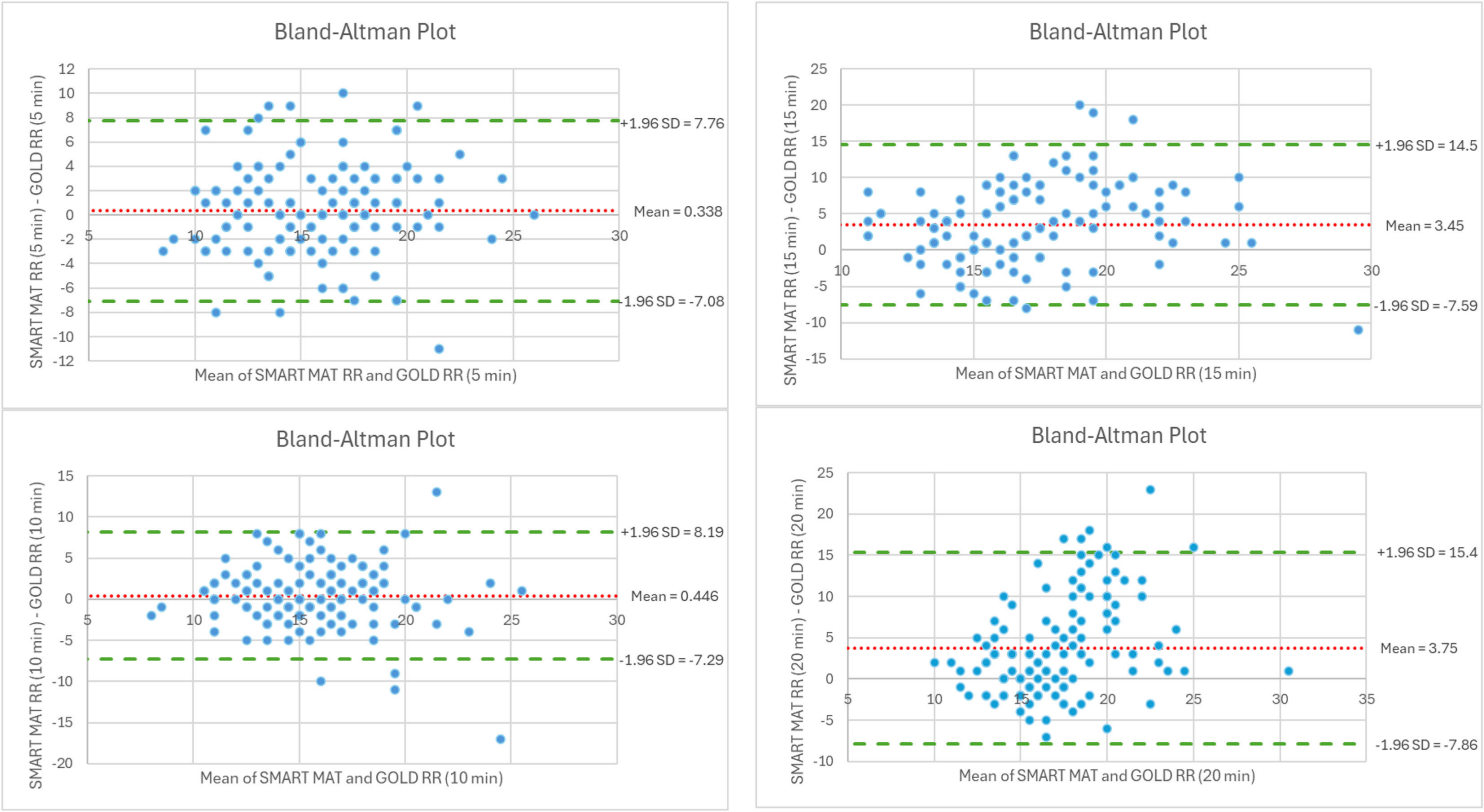
Compiled Bland-Altman plot for respiratory rate (5^th^, 10^th^, 15^th^ & 20^th^ minute).

Analysis of RR measurements (**Table 2**) revealed a distinct divergence in accuracy between stationary and mobile conditions. During the stationary phases, the sitting SMART MAT demonstrated high concordance with clinical standards, as evidenced by a low MAE of 2.85-2.86 breaths/min. No statistically significant differences were observed at the 5^th^ minute (p=0.310) or 10^th^ minute (p=0.200), with small effect sizes (η^2^: 0.008 to 0.013), MAPE values remaining within the good range (19.2% to 20.1%). Conversely, wheelchair motion impacted sensor performance during the 15^th^ and 20^th^ minutes. RR readings showed a statistically significant increase (p < 0.001) compared to the clinical standard, with large effect sizes (η^2^ > 0.275). The magnitude of error increased as the wheelchair moved, with the MAE rising from 5.03 to 5.21 breaths/min. The Bland-Altman analysis (**Figure 2**) confirms this trend; while the LoA widened during motion, the magnitude of error remains clinically acceptable for continuous, non-diagnostic trend monitoring. *Post-hoc* Bonferroni correction confirmed significant difference (*p>* 0.001) during these mobile intervals, suggesting that while the SMART MAT is reliable under static conditions, motion artifacts introduce considerable measurement error.

**Table 2:** Descriptive and inferential statistics of respiratory rate.

Time periods	Median (breaths/min)	IQR (breaths/min)	*p*-value	*F*-value	η^2^	MAE	MAPE (%)	*pBonf*
Time 1 (5th minute)								
SMART MAT	16.0	13.3-18.0	0.310	1.04	0.008	2.86	20.1	0.310
Clinical Reference	16.0	12.0-18.0						
Time 2 (10th minute)								
SMART MAT	16.0	14.0-18.0	0.200	1.66	0.013	2.85	19.2	0.200
Clinical Reference	15.0	13.0-17.0						
Time 3 (15th minute)								
SMART MAT	18.0	15.0-22.8	< 0.001*	48.84	0.275	5.21	38.7	< 0.001*
Clinical Reference	15.0	13.0-17.0						
Time 4 (20th minute)								
SMART MAT	17.0	15.0-23.8	< 0.001*	52.16	0.288	5.03	47.6	< 0.001*
Clinical Reference	15.0	13.0-17.0						

IQR, interquartile range 25%-75%; n^2^, effect size; MAE, mean absolute error; MAPE, mean absolute percentage error; Clinical Reference, manual counting of respiratory rate, *: denotes *p* <.05 represents a statistical significance.

**Figure 3:**
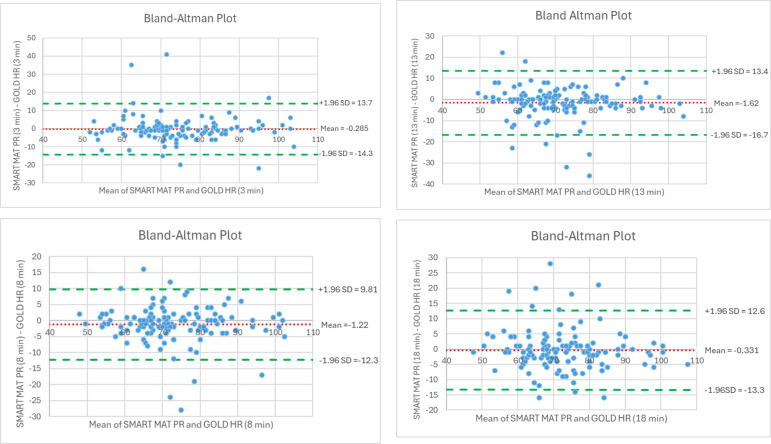
Compiled Bland-Altman plot for heart/pulse rate (3^rd^, 8^th^, 13^th^ & 18^th^ minute).

Descriptive statistics and MAE values for HR across all time intervals are detailed in **Table 3**. The sitting SMART MAT demonstrated consistently high accuracy and clinical agreement, maintaining a low MAE between 3.67 and 4.81 bpm and with MAPE values below 7% across all static and mobile conditions. Median SMART MAT values were slightly lower than the clinical reference standard, with an underestimation of 0.5-2.0 bpm. Statistical analysis revealed that variances at the 3^rd^ and 18^th^ minutes were not significant (p = 0.569-0.651), with negligible effect sizes (η^2^ ≤ 0.003). While the 8^th^- and 14^th^-minute points showed statistically significant differences (p = 0.014 to 0.018), the associated effect sizes remained small (η^2^ = 0.043 to 0.045). Bland-Altman analysis (**Figure 3**) confirmed a slight negative mean bias of -0.285 to -1.62 bpm, with 95% limits of agreement (LoA) of -16.7 to 13.7 bpm. The tight clustering of data points within these limits indicates the device maintained robust performance throughout the study protocol.

**Table 3:** Descriptive and inferential statistics of heart/pulse rate.

Time periods	Median (bpm)	IQR (bpm)	*p*-value	*F*-value	η^2^	MAE	MAPE (%)	*pBonf*
Time 1 (3rd minute)								
SMART MAT	71.0	66.0-81.8	0.651	0.206	0.002	4.16	6.08	0.651
Clinical Reference	72.0	66.0-82.0						
Time 2 (8th minute)								
SMART MAT	70.0	64.0-80.0	0.014*	6.14	0.045	3.67	4.97	0.014*
Clinical Reference	72.0	65.3-81.8						
Time 3 (13th minute)								
SMART MAT	69.0	62.0-78.0	0.018*	5.77	0.043	4.81	6.75	0.018*
Clinical Reference	70.5	64.0-78.8						
Time 4 (18th minute)								
SMART MAT	70.0	64.3-79.0	0.569	0.327	0.003	4.42	6.42	0.569
Clinical Reference	71.0	64.3-80.0						

bpm , beats per minute; IQR , interquartile range 25%-75%, η^2^ , effect size, MAE , mean absolute error, MAPE , mean absolute percentage error, Clinical Reference , Schiller CardioVit AT-1 G2 Electrocardiogram, *: denotes p <.05 represents a statistical significance.

**Figure 4:**
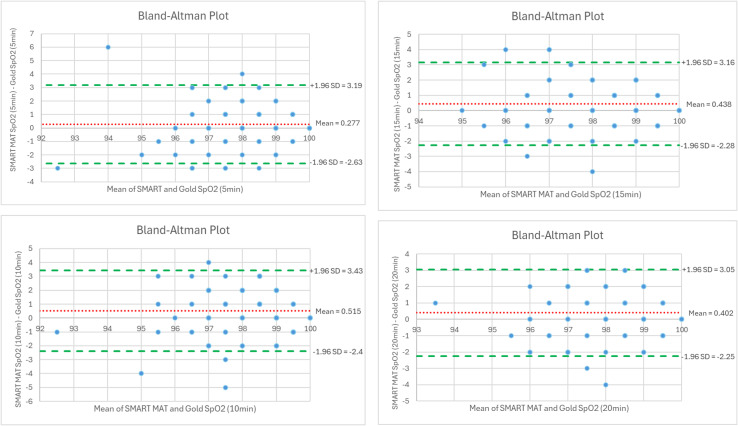
Compiled Bland-Altman plot for SpO_2_ (5^th^, 10^th^, 15^th^ & 20^th^ minute).

Descriptive and inferential statistics for SpO_2_ are summarized in **Table 4**. Both the clinical reference standard and the SMAT MAT showed consistent median values of 98%-99% throughout the study, with overlapping IQR. Although a repeated-measures ANOVA identified statistically significant differences across all time intervals (p < 0.05), the effect sizes were small at the 5^th^ minute (η^2^ = 0.034) and became medium in subsequent intervals (η^2^ = 0.073 to 0.108). Despite these statistical variances, the SMART MAT demonstrated exceptional clinical reliability. The MAE and MAPE remained remarkably low, ranging from 1.08% to 1.21% and 1.11 to 2.85%, respectively, across all measured intervals. Bland-Altman analysis (**Figure 4**) revealed a slight positive mean bias ranging from 0.277% to 0.515%, with a 95% LoA from -2.63% to 3.43%. These results indicate that while the sitting SMART MAT tends to slightly overestimate oxygen saturation, the deviation falls within the standard ±2%-3% clinical tolerance threshold typical of conventional pulse oximeters, confirming its practical utility.

**Table 4:** Descriptive and inferential statistics for oxygen saturation.

Time periods	Median (%)	IQR (%)	*p*-value	*F*-value	η^2^	MAE	MAPE (%)	*pBonf*
Time 1 (5th minute)								
SMART MAT	98	97.0-99.0	0.035*	4.53	0.034	1.08	1.11	0.035*
Clinical Reference	98	97.0-99.0						
Time 2 (10th minute)								
SMART MAT	99	98.0-99.0	< 0.001*	15.7	0.108	1.19	1.22	< 0.001*
Clinical Reference	98	97.0-99.0						
Time 3 (15th minute)								
SMART MAT	99	97.0-99.0	< 0.001*	13.0	0.092	1.08	1.11	< 0.001*
Clinical Reference	98	97.0-99.0						
Time 4 (20th minute)								
SMART MAT	99	98.0-99.0	0.002*	10.2	0.073	1.21	2.85	0.002*
Clinical Reference	98	97.0-99.0						

IQR, interquartile range 25%-75%; η^2^, effect size; MAE, mean absolute error; MAPE, mean absolute percentage error; Clinical Reference, NellcorTM pulse oximeter, *: denotes p <.05 represents a statistical significance.

**Figure 5:**
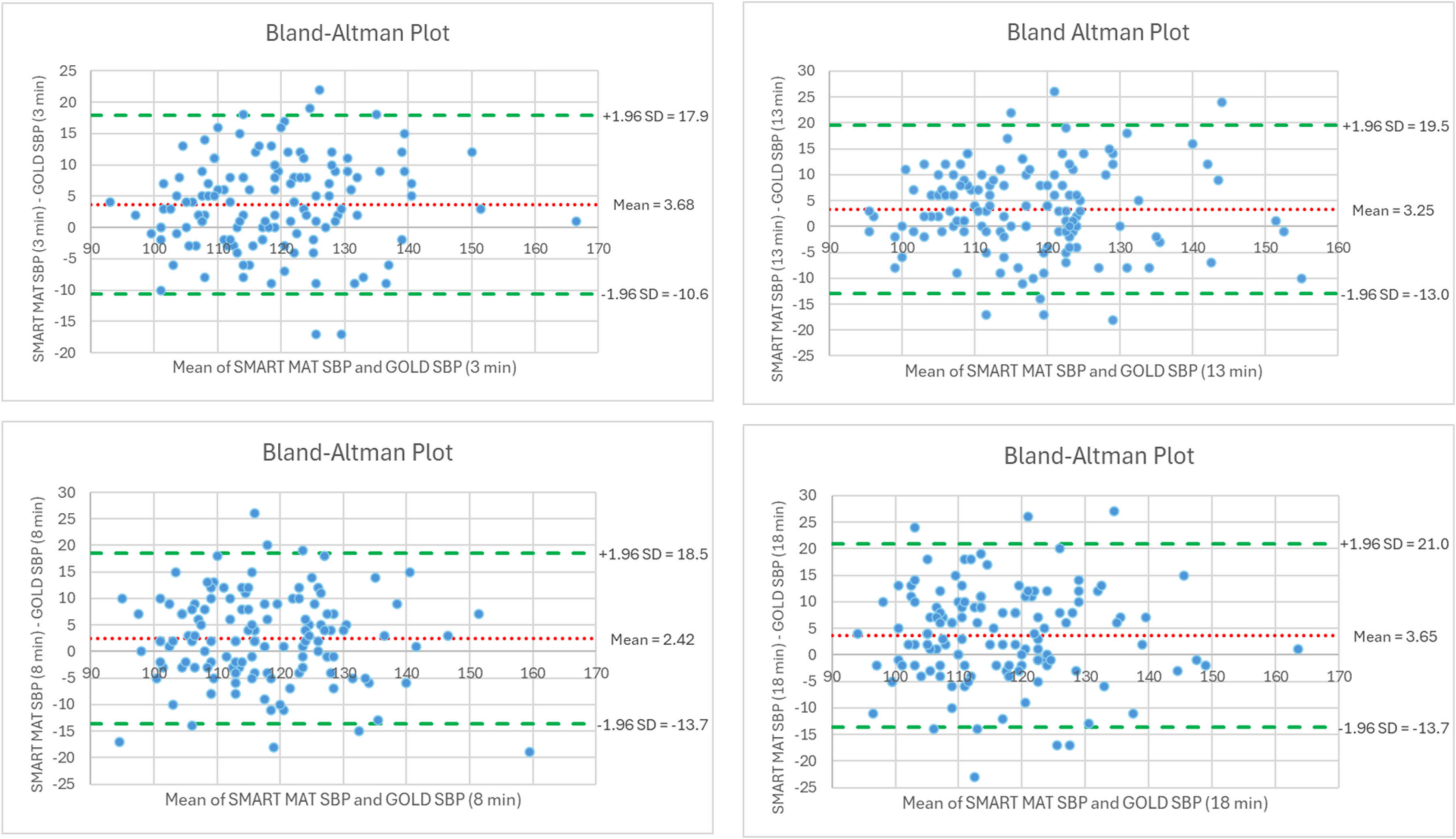
Compiled Bland-Altman Plot for systolic blood pressure (3^rd^, 8^th^, 13^th^ & 18^th^ minute).

**Figure 6:**
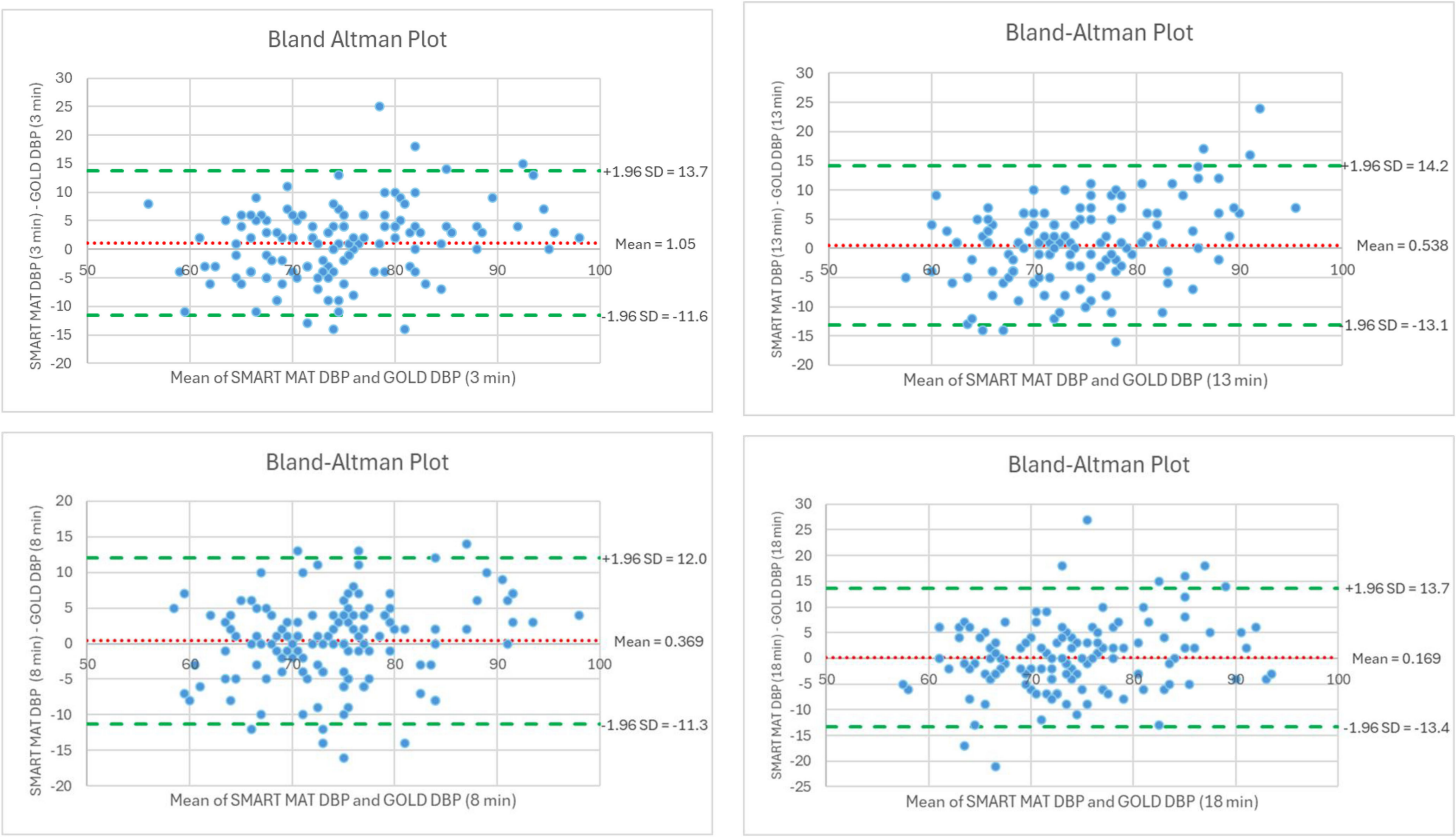
Compiled Bland-Altman plot for diastolic blood pressure (3^rd^, 8^th^, 13^th^ & 18^th^ minute).

The comparative analysis of blood pressure measurements revealed mixed performance patterns for SBP (**Table 5**) and DBP (**Table 6**). The sitting SMART MAT consistently overestimated SBP by a mean of 2–4 mmHg relative to the clinical reference standard, yielding an MAE of 6.48-7.51 mmHg. Repeated measures ANOVA confirmed that these differences were statistically significant across all intervals (p ≤ 0.001), with effect sizes ranging from moderate to large. While the central error indicates solid aggregate accuracy, Bland-Altman plots (**Figure 5**) showed wider LoA that occasionally exceeded the rigorous ±5 mmHg clinical threshold, suggesting greater individual variability alongside a steady mean bias of 2.42 to 3.68 mmHg. In contrast, DBP measurements demonstrated superior stability and tighter agreement. The MAE remained lower (4.65 to 5.46 mmHg), and non-statistically significant agreement (p = 0.064 to 0.781) was observed with minimal variance and negligible effect sizes (η^2^ < 0.01). Blant-Altman analysis (**Figure 6**) further corroborated this, with mean bias for DBP narrowing from 1.05 mmHg to a negligible 0.169 mmHg by the end of the protocol. Collectively, these findings suggest that while the SMART MAT is highly reliable for DBP readings, individual SBP readings exhibit greater variance, positioning the current SBP function as a continuous trend monitor rather than a definitive diagnostic replacement.

**Table 5:** Descriptive and inferential statistics for systolic blood pressure.

Time periods	Median (mmHg)	IQR (mmHg)	*p*-value	*F*-value	η^2^	MAE	MAPE (%)	*pBonf*
Time 1 (3rd minute)								
SMART MAT	120	111–129	< 0.001*	33.3	0.034	6.48	5.57	< 0.001*
Clinical Reference	117	107-124						
Time 2 (8th minute)								
SMART MAT	117	110-127	< 0.001*	11.3	0.108	6.82	5.96	< 0.001*
Clinical Reference	115	106-124						
Time 3 (13th minute)								
SMART MAT	117	110-125	< 0.001*	20.1	0.092	6.95	6.73	< 0.001*
Clinical Reference	115	106-123						
Time 4 (18th minute)								
SMART MAT	117	109-124	< 0.001*	22.2	0.073	7.51	6.73	< 0.001*
Clinical Reference	114	104-122						

IQR, interquartile range 25%-75%; η^2^, effect size; MAE, mean absolute error; MAPE, mean absolute percentage error; Clinical Reference, Bokang LCD Non-Mercury Sphygmomanometer, *: denotes *p* <.05 represents a statistical significance.

**Table 6:** Descriptive and inferential statistics for diastolic blood pressure.

Time periods	Median (mmHg)	IQR (mmHg)	*p*-value	*F*-value	η^2^	MAE	MAPE (%)	*pBonf*
Time 1 (3rd minute)								
SMART MAT	74.0	69.0-81.0	0.064	3.49	0.034	5.10	1.11	0.064
Clinical Reference	74.0	68.0-78.0						
Time 2 (8th minute)								
SMART MAT	73.5	68.3-79.0	0.480	0.503	0.108	4.65	1.22	0.480
Clinical Reference	73.0	69.0-78.0						
Time 3 (13th minute)								
SMART MAT	73.0	67.0-80.0	0.379	0.778	0.092	5.46	1.11	0.379
Clinical Reference	73.0	70.0-79.0						
Time 4 (18th minute)								
SMART MAT	73.0	67.0-79.0	0.781	0.078	0.073	5.17	2.85	0.781
Clinical Reference	74.0	68.0-78.0						

IQR, interquartile range 25%-75%; η^2^, effect size; MAE, mean absolute error; MAPE, mean absolute percentage error; Clinical Reference, Bokang LCD Non-Mercury Sphygmomanometer, *: denotes *p* <.05 represents a statistical significance.

## 4 Discussion

This study is the first to systematically assess the accuracy and validity of the sitting SMART MAT compared with established clinical reference standards. The study’s findings suggest that this integrated multimodal platform yields measurements highly comparable to those of traditional methods, especially under stationary conditions, thereby validating its utility as a continuous monitoring tool.

The study cohort comprised a balanced representation of healthy adult males and females. The sample exhibited significant anthropometric variations consistent with gender-based dimorphism: males showed significantly higher means for height, weight, muscle mass, fat-free mass, and total body water percentage, whereas females showed significantly higher body fat percentage (p < 0.001; r = -0.953 to 0.751). Although there were significant differences in height and weight, the overall BMI did not differ significantly between genders (p = 0.066, r = -0.188), aligning with the World Health Organization guidelines (WHO, 2004). However, utilizing BMI as the sole metric of physical health, despite its ease of calculation, fails to account for variations in body composition (Muscogiuri et al., 2023). While the observed gender differences in body fat percentage and muscle mass are physiologically normal (Janssen et al., 2000), male and female participants maintained acceptable health parameters. The cohort thus provides a robust baseline for healthy adults, though future studies may extend validation to broader clinical demographics.

Analysis of RR measurements revealed strong concordance between the sitting SMART MAT and the clinical reference standard during stationary phases. The differences were within the accepted measurement bias of ±3 breaths per minute when stationary (Chan et al., 2022). However, wheelchair motion introduced significant variance and higher MAE values at the 15^th^ and 20^th^ minutes, indicating a statistically significant difference with a larger effect size (η^2^ = 0.275-0.288, p < 0.001). These findings align with prior literature indicating that activity artifacts and gross physical movement pose challenges for RR monitoring using BCG and pressure-variation analysis (Chokalingam et al., 2026). Additionally, the SMART MAT’s algorithm-based calculations are susceptible to external disturbances. Clinical reference-standard measurements via chest wall observation are also vulnerable to participant awareness, observation methods, and value bias (Kallioinen et al., 2021). Nevertheless, because normal resting RR typically ranges between 12 and 20 breaths/min, these deviations remain clinically acceptable. Therefore, the device is reliable when the user is stationary, and discrepancies can be resolved by allowing the mat a few seconds to recalibrate after movement.

The SMART MAT demonstrated robust performance in HR monitoring, with MAE values consistently remaining below 5 bpm across all time points. According to the International Electrotechnical Commission (2011), this level of accuracy is acceptable for clinical use. ANOVA results indicated that variances at the 3^rd^ and 19^th^ minutes were not statistically significant (p = 0.569 to 0.651, η^2^ ≤ 0.003). Although significant differences were noted at the 8^th^ and 13^th^ minutes (p < 0.05), the effect sizes were small (η^2^ = 0.043 to 0.045). The observed underestimation by 0.5 to 2.0 bpm is consistent with existing literature, which indicates the BCG morphology varies based on posture, position, and individual physiology (Sadek et al., 2019). Furthermore, user movement, such as coughing or positional shifts, can produce artifacts and affect reading amplitude (Fajkus et al., 2017). Since BCG signals are most stable near the chest level (Zhu et al., 2014), the seated posture introduces minor variations, but the measurement remains highly reliable as an alternative to clinical standards.

Both the clinical reference standard and the sitting SMART MAT yielded consistent median SpO_2_ values between 98% and 99% throughout the protocol, with overlapping IQR. Although the repeated-measures ANOVA identified statistically significant differences across all time intervals, the effect sizes remained small to medium (η^2^ = 0.034-0.108). The narrow physiological range of SpO_2_, typically 95% to 100% in healthy individuals (Hafen and Sharma, 2026), makes it sensitive to statistical detection even for clinically negligible deviations. The low MAE (1.08% to 1.21%) confirms high forecast accuracy. The minor discrepancies observed may be attributed to body composition or movement artifacts, which can influence optical recordings (León-Valladares et al., 2024). Overall, the deviations remain within the 2-4% margin of error expected for conventional pulse oximetry (Torp et al., 2026).

Comparative analysis revealed divergent performance patterns between SBP and DBP. The sitting SMART MAT paired device consistently overestimated SBP by 2–4 mmHg across time intervals. These differences were statistically significant (p < 0.001) and had moderate-to-large effect sizes. Despite this, the values fall within the “very accurate” band of the British Hypertension Society (O’Brien et al., 2002). This systematic error may arise from the oscillometric technique used in the paired cuff (Meidert and Saugel, 2017) rather than from the fiberoptic sensors themselves, or from inter-arm differences during measurement (Orme et al., 1999). Although the broad normative SBP range (Jones et al., 2025) accommodates these differences, caution is warranted to avoid misdiagnosis in borderline cases. While the central accuracy is strong, the Bland-Altman LoA occasionally exceeded the clinically acceptable ±5 mmHg threshold (Kim et al., 2014), indicating greater individual variability. Consequently, in its current iteration, the SBP function is best positioned as a continuous, passive screening tool to detect broad blood pressure trajectories rather than as a definitive diagnostic replacement. The observed upward trend in SBP variance over time may have reflected minor postural adjustments or slight shifts in seating mechanics as the session progressed, suggesting an area where future algorithmic refinement could improve stability. In contrast, DBP measurements demonstrated remarkable concordance with the clinical standard. Variance was minimal (F-value ≤ 0.778, p > 0.05) across all intervals after the 3^rd^ minute. MAE values for DBP ranged from 4.65 to 5.46 mmHg, indicating high accuracy. The Bland-Altman bias narrowed over time to just 0.169 mmHg, indicating that the paired device recalibrated well after movement. These findings confirm the feasibility of the SMART MAT for DBP measurement.

### 4.1 Clinical implications

These findings suggest that the sitting SMART MAT is a viable, non-invasive system for sub-acute and community settings such as nursing homes, rehabilitation centers, and home environments. While the vast majority of the existing non-contact sensors are engineered exclusively for mattresses, validating this technology for a seated posture addresses a critical clinical need. Prolonged bed rest in elderly populations is strongly associated with physical deconditioning, pressure injuries, and pulmonary complications. By facilitating continuous monitoring in chairs or wheelchairs, the SMART MAT encourages safe patient mobilization without sacrificing clinical surveillance. Beyond eliminating the need for skin contact or restrictive attachments, the continuous, real-time streaming of physiological data to smart devices represents a major step forward in smart physiological monitoring. By providing caregivers with instant access to vital sign displacements without adding to the patient’s physical or psychological burden, the system creates a truly passive monitoring environment. By reducing the reliance on manual observations, the system minimizes the labor-intensive workload of healthcare professionals and supports the early detection of clinical deterioration. However, the systematic overestimation and statistical variance in SBP highlights the need to continue refining the paired BP monitoring devices before unrestricted clinical deployment.

### 4.2 Limitations

Several limitations must be considered when interpreting these findings. Crucially, while this study evaluated the system against established, non-invasive clinical methods (e.g., sphygmomanometer, standard pulse oximetry), it did not utilize true, invasive physiological gold standards (such as intra-arterial catheters or arterial blood gas analysis), as deploying invasive lines in a healthy cohort was ethically and practically unfeasible. Secondly, the study utilized manual observation to record respiratory rate (RR) rather than an automated respiratory belt. Although this approach aligns with standard clinical practice among nursing staff in general wards, manual observation is inherently susceptible to human variation and observer bias. Thirdly, the evaluation took place in a highly controlled environment, utilizing linear wheelchair movement on a flat, level surface. These conditions do not fully replicate the varied terrain, slopes, and unpredictable physical movements encountered in actual community or clinical settings. To maintain experimental control during data collection, participants were required to remain still and avoid speaking or eating. Furthermore, individuals with known arrhythmias, such as atrial fibrillation and flutter, were excluded. As these conditions are highly prevalent in the geriatric demographic and because the device relies on detecting regular ballistocardiographic waves, this exclusion limits the generalizability of our immediate findings. Future algorithmic training and validation studies must specifically address irregular rhythms. Finally, the validation protocol was limited to 20 minutes per participant. Consequently, the long-term reliability, skin contact, and sensor stability of the sitting SMART MAT over extended monitoring periods remain to be investigated in future studies. Future research should conduct clinical trials involving diverse, high-risk, and geriatric populations to ensure the device’s reliability among individuals with active cardiorespiratory conditions. Additionally, incorporating artificial intelligence (AI) with adaptive calibration algorithms can optimize the processing of motion-induced artifacts, tailor physiological thresholds, and minimize false-positive alarms, thereby enhancing clinical utility.

## 5 Conclusion

This study demonstrates the clinical validity and accuracy of the sitting SMART MAT as an integrated multimodal platform for continuous vital-sign monitoring under both static and mobile conditions. Furthermore, by accommodating a sitting position, a far more natural and functional posture than bed rest, the sitting SMART MAT accurately captures physiological fluctuations in realistic, everyday scenarios. Crucially, while most existing sensor arrays target bed-bound patients, individuals who can sit upright routinely are far less prone to the secondary complications associated with prolonged bed rest and immobility. Therefore, this capability enables continuous, non-invasive monitoring without restricting patient movement, thereby demonstrating its potential for real-world application. By eliminating the need for restrictive chest attachments for cardiovascular and respiratory tracking with centrally aggregating peripheral data, the SMART MAT facilitates seamless, real-time physiological surveillance in sub-acute, nursing home, and home telehealth environments. These capabilities substantially reduce the workload of caregivers or healthcare professionals while supporting the early detection of clinical deterioration, serving as a vital transitional step toward fully passive physiological surveillance.

## Data Availability

The raw data supporting the conclusions of this article will be made available by the corresponding author upon reasonable request.
