# Supplementary material for: Seamless vital sign monitoring with fiber optic sensing technology: validating the sitting SMART MAT for community health in static and mobile conditions
Source: Front Physiol. 2026 Jul 23;17:1875461. doi: 10.3389/fphys.2026.1875461 (PMC13442818; doi:10.3389/fphys.2026.1875461)
Supplement: Supplementary file 1 [file SupplementaryFile1.docx]

Supplementary Material

Supplementary Figure 1: The study set-up of the stting SMART MAT on the standard wheelchair with a standardized seat cushion (5 cm thickness) above

# Supplementary Figure 2a: the form factor of the sitting SMART MAT

Supplementary Figure 2b: the form factor of the sitting SMART MAT

## Supplementary Figure 3: Set up of participants with attachments

Supplementary Figure 4: Timeline of data collection

*Note. SBP = Systolic Blood Pressure, DBP = Diastolic Blood Pressure and SpO2 = Oxygen Saturation*
